# Response of forage nutrient storages to grazing in alpine grasslands

**DOI:** 10.3389/fpls.2022.991287

**Published:** 2022-11-01

**Authors:** Xinjie Zha, Yuan Tian, Ouzhu, Gang Fu

**Affiliations:** ^1^Xi'an University of Finance and Economics, Xi'an, China; ^2^Lhasa Plateau Ecosystem Research Station, Key Laboratory of Ecosystem Network Observation and Modeling, Institute of Geographic Sciences and Natural Resources Research, Chinese Academy of Sciences, Beijing, China; ^3^Agriculture and Animal Husbandry Comprehensive Service Center of Gamba County, Shigatse, China

**Keywords:** biodiversity, seasonal grazing, classification management, altitude distribution, forage, nutrient quality, Tibetan Plateau (TP)

## Abstract

Forage nutrient storages can determine livestock size and husbandry development. There is insufficient research on the response of forage nutrient storages to grazing and related driving mechanisms in alpine grasslands, especially on the Tibetan Plateau. This study conducted a grazing experiment in three alpine grassland sites along an elevation gradient (two warm-season pastures and one cold-season pasture; two alpine steppe meadow sites and one alpine meadow) of Northern Tibet. Different types of alpine grassland ecosystems, at least for forage nutrient storages, may have different responses to grazing. Warm-season grazing significantly reduced crude protein (CP) storage, acid detergent fiber (ADF) storage, and neutral detergent fiber (NDF) storage of high-quality forage by 53.29, 63.82, and 63.26%, respectively, but cold-season grazing did not significantly alter the CP, ADF and NDF storages of high-quality forage. Warm-season grazing significantly reduced CP, ADF, NDF, crude ash (Ash), ether extract (EE) and water-soluble carbohydrate (WSC) storages of the plant community by 46.61, 62.47, 55.96, 64.94, 60.34, and 52.68%, and forbs by 62.33, 77.50, 73.69, 65.05, 57.75, and 62.44% in the alpine meadow site but not the alpine steppe meadow site, respectively. Plant species and phylogenetic diversity had different relationships with forage nutrient storages. The elevation distribution of forage nutrient storages under fencing conditions were different from those under grazing conditions. Therefore, cold-season grazing can have lower negative effects on forage nutrient storages than warm-season grazing. Combined plant species with phylogenetic diversity and composition can be better in predicting forage nutrient storages. Grazing can restructure the elevation distribution of forage nutrient storages in alpine grasslands.

## Introduction

Grasslands, including alpine meadows, alpine steppes, temperate steppes, prairies, and other types, are essential parts of global terrestrial ecosystems, and widely distributed all over the world (Reynolds et al., 2015; Fu and Shen, 2022; Wang et al., 2022). Grassland ecosystems are not only the habitat of grassland wild animals and plants, but also the food source of grazing livestock, the resource of high-quality livestock husbandry development, and one of the important sources of livelihood of herders (Wu and Fu, 2018; Piao et al., 2019; Fu and Sun, 2022). Grazing, as one of the main human activities and land-utilization type in grassland ecosystems, is always used as an external disturbance factor to explore its influence on the structures and functions of grassland ecosystems on different scales of time and space (Milchunas et al., 1995; Dlamini et al., 2016; Gao and Carmel, 2020). However, there are still some debates which can be further reconsidered. First, the debate on the relative influence magnitude of cold-season grazing and warm-season grazing on ecosystem structures and functions of grasslands is still on-going. Some studies have displayed that cold-season grazing can have greater negative effects on ecosystem structures and functions of grasslands than warm-season grazing (Fu et al., 2012). By contrast, other studies have discovered that warm-season grazing can have greater negative influences on ecosystem structures and functions of grasslands than cold-season grazing (Sun et al., 2021; Zhang and Fu, 2021). Second, both species diversity and phylogenetic diversity are essential parts of biodiversity; but, there is still a debate on which one, species diversity or phylogenetic diversity, has closer correlations with ecosystem structures and functions of grasslands (Wang et al., 2021b). Moreover, compared to phylogenetic diversity, more studies have explored the correlations between species diversity and ecosystem structures and functions of grasslands (Wu et al., 2014b; Wang et al., 2021a). Third, α- and β-diversity are different aspects of biodiversity, and there is also still debate on which one, α- or β-diversity, has closer correlations with ecosystem structures and functions of grasslands (Fu et al., 2021; Wang et al., 2021a). Moreover, compared to β-diversity, more studies have explored the correlations between α-diversity and ecosystem structures and functions of grasslands (Yu et al., 2019; Zhang et al., 2021). Fourth, the spatial homogenization for ecosystem structures and functions may portend the loss of ecosystem structures and functions, whereas the spatial heterogenization for ecosystem structures and functions may portend the increase of ecosystem structures and functions in grassland ecosystems. There is still debate on whether grazing can homogenize or heterogenize spatial distribution of ecosystem structures and functions of grasslands (Fu et al., 2022; Zhang and Fu, 2022). Therefore, more studies are needed to improve adaptive management and use of grassland ecosystems under future global change.

The Tibetan Plateau is one of the regions with widely distributed grassland ecosystems, and thus many earlier studies have tried to explore grazing impacts on ecosystem structures and functions in alpine grasslands and related driving mechanisms (Xiong et al., 2016; Han et al., 2022). These earlier studies can provide an essential scientific foundation for adaptive grazing management of alpine grasslands. However, aside from the debates mentioned above, there are still some debates which can be further explored. For example, as is well known, forage nutrient storages, as key indicators of grassland quality and livestock capacity, likely play vital roles in the balance of forage-livestock, the protection of biodiversity, livestock product quality and amount, and even income of herders (Pontes et al., 2007; Sasaki et al., 2012; Elgersma and Søegaard, 2016). Compared to forage nutrient quality, biodiversity, plant production, and soil variables, only a few studies have tried to investigate the forage nutrient storages in alpine grasslands on the Tibetan Plateau (Xiong et al., 2016). This phenomenon implies that there are still great uncertainties in our knowledge of ecosystem quality and nutrient carrying capacity under grazing conditions in alpine grasslands on the Tibetan Plateau. Therefore, more studies are needed to better investigate the grazing impacts on forage nutrient storages and in turn grassland quality on the Tibetan Plateau.

In this study, a field experiment was conducted in three alpine grassland sites. The main objectives of this research were to investigate whether (1) cold-season grazing had greater impacts on forage nutrient storages than warm-season grazing; (2) plant phylogenetic diversity had different correlations with forage nutrient storages than plant species diversity; (3) plant α-diversity had different correlations with forage nutrient storages than plant β-diversity; and (4) grazing can alter spatial distributions of forage nutrient storages in alpine grasslands. First, expanding on a few earlier studies (Fu et al., 2021; Sun et al., 2021; Zhang and Fu, 2021), we hypothesized that the response of forage nutrient storages to cold-season grazing was lower compared to warm-season grazing (H1). Second, extending from a few earlier studies (Fu et al., 2021; Sun et al., 2021; Wang et al., 2021a), we hypothesized that plant species diversity and plant α-diversity had different correlations with plant phylogenetic diversity and plant β-diversity, respectively (H2). Third, following a few earlier studies (Fu et al., 2022; Zhang and Fu, 2022), we hypothesized that grazing could reconstruct the elevation patterns for forage nutrient storages (H3).

## Materials and methods

### Experimental design

The field experiment was conducted in three alpine grassland sites (A: 91.07 °E, 30.50 ° N, 4313 m; B: 91.06 °E, 30.52 ° N, 4513 m; C: 91.05 °E, 30.53 ° N, 4693 m) located at the Damxung County, Lhasa City, Tibet Autonomous Region, China, in July, 2008 (Sun et al., 2021; Zhang and Fu, 2021). The sites A and B are alpine steppe meadows (dominant species are *Carex atrofusca, Stipa capillacea* and *Kobresia pygmaea*), whereas the site C is an alpine meadow (dominant species is *Kobresia pygmaea*). Site A is originally a cold-season pasture, whereas sites B and C are originally warm-season pasture. At each site, there was an approximate 0.02 × 0.02 km enclosure since July 2008. There were two experimental treatments (fencing and free grazing treatments) for each of the three sites, and there was a total of six experimental treatments. There were generally domestic sheep and yak around the three sites (Sun et al., 2021). Mean annual temperature was about 3.24, 1.96, and 0.88 °C as measured from 1982 to 2020 at site A, B, and C, respectively. Mean annual precipitation was about 450.70 mm, 459.38 mm, and 466.22 mm during the 1982–2020 time period at site A, B, and C, respectively. The precipitation was about 397.5 mm, 407.6 mm, and 416.5 mm during June–September in 2000–2018 at site A, B, and C, respectively (Sun et al., 2022). Mean annual radiation was about 6619.54 MJ m^−2^, 6649.00 MJ m^−2^, and 6679.96 MJ m^−2^ during 2000–2020 at site A, B, and C, respectively.

### Plant and soil sampling and analyses

In August 2019, plant community investigation (species richness, community coverage and height, species coverage and height), aboveground biomass for each species, and soil sampling at 0–10 cm depth were conducted/collected under both fencing and grazing conditions at each site. Each one of the six treatments had four replicates, and each quadrat size was a 0.50 × 0.50 m. The quadrats were spaced at least 5–6 m apart. The aboveground portion of each species was clipped at ground level using scissors or wallpaper cutters, and put into envelopes by species. Collected plant samples were first oven-dried for 48 h and weighted for each species, and then used to measure crude protein (CP), acid detergent fiber (ADF), neutral detergent fiber (NDF), crude ash (Ash), ether extract (EE) and water-soluble carbohydrates (WSC) concentration for high-quality forages (i.e., *Carex atrofusca, Stipa capillacea* and *Kobresia pygmaea*) and forbs, respectively (Sun et al., 2020) by Shandong Zhong Sublimation Inspection Certification Testing Co. LTD (Shandong Province, China). After plant sample collection, soil augers (about 0.037 m in diameter) were used to collect soil samples within each 0.50 × 0.50 m quadrat. Collected soil samples were stored at −20 °C before analyses, and then used to measure soil organic carbon (SOC), total nitrogen (TN), total phosphorus (TP), ammonium nitrogen (${\text{NH}}_{4}^{+}$-N), nitrate nitrogen (${\text{NO}}_{3}^{-}$-N), available phosphorus (AP) and pH (Sun et al., 2021; Zhang and Fu, 2021) by Xilin Gol League Baisheng Biotechnology Co., LTD (Inner Mongolia Autonomous Region, China).

### Statistical analyses

We calculated the storages of crude protein, acid detergent fiber, neutral detergent fiber, crude ash, ether extract, and water-soluble carbohydrates by measuring the concentration of crude protein, acid detergent fiber, neutral detergent fiber, crude ash, ether extract, and water-soluble carbohydrates in aboveground biomass for high-quality forages and forbs, respectively. We then calculated the storages of crude protein, acid detergent fiber, neutral detergent fiber, crude ash, ether extract, and water-soluble carbohydrates for plant community using the weight of high-quality forages and forbs biomass. We calculate the ratio of SOC to TN (C:N), SOC to TP (C:P), TN to TP (N:P), the sum of ${\text{NH}}_{4}^{+}$-N and ${\text{NO}}_{3}^{-}$-N to AP (available N:P), and ${\text{NH}}_{4}^{+}$-N to ${\text{NO}}_{3}^{-}$-N (${\text{NH}}_{4}^{+}$-N: ${\text{NO}}_{3}^{-}$-N). We calculated species α-diversity (SR: species richness, Shannon, Simpson, and Pielou indexes) and phylogenetic α-diversity (PD: phylogenetic diversity, MNTD: mean nearest taxon distance), species β-diversity (βBray) and phylogenetic β-diversity (βMNTD) for plant community, high-quality forages and forbs, respectively, from the vegan and/or picante packages. We compared the differences of CP, ADF, NDF, Ash, EE, and WSC storages between grazing and fencing conditions using *T*-test from the stats package, respectively. We analyzed the differences for the data matrix of CP, ADF, NDF, Ash, EE, and WSC storages between grazing and fencing conditions using the adonis2 function from the vegan package. We analyzed the correlations between variables related to nutrient storages, and environmental variables (including soil variables, plant diversity variables) using Random Forest Model from the randomForest and rfPermute packages. All the statistical analyses were performed by R 4.1.2.

## Results

### Impacts of grazing on the crude protein storage, acid detergent fiber storage, neutral detergent fiber storage, crude ash storage, ether extract storage, water-soluble carbohydrates storage and nutrient storages

**Figure 1:**
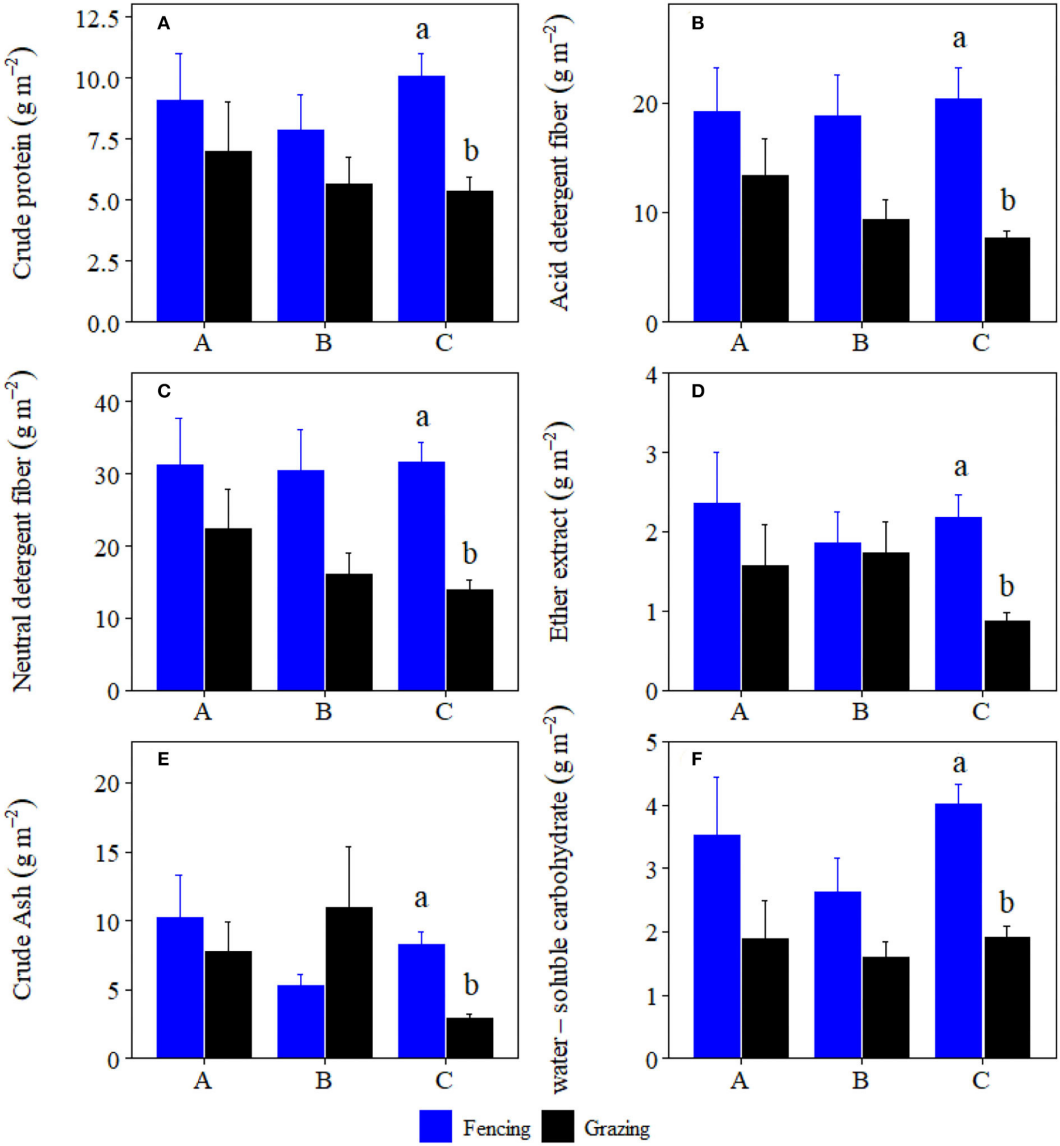
Comparisons of plant community **(A)** crude protein, **(B)** acid detergent fiber, **(C)** neutral detergent fiber, **(D)** ether extract, **(E)** crude ash, and **(F)** water-soluble carbohydrate storages (mean ± SE) between the fencing and grazing conditions in three alpine grasslands located at site A–C, respectively. Different letters indicated significant difference for nutrition storages between the fencing and grazing conditions at *p* < 0.05 level.

**Figure 2:**
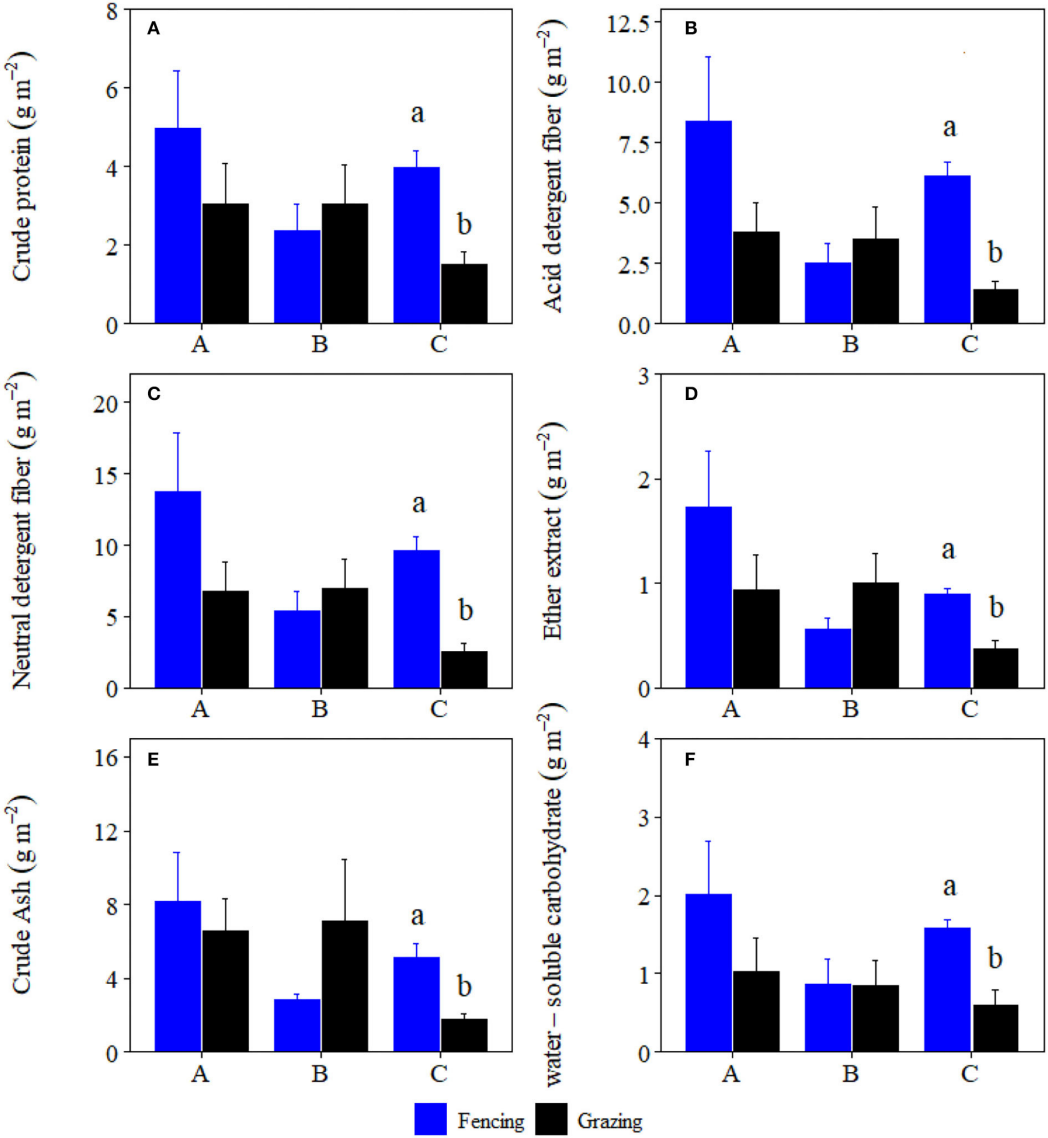
Comparisons of forbs **(A)** crude protein, **(B)** acid detergent fiber, **(C)** neutral detergent fiber, **(D)** ether extract, **(E)** crude ash, and **(F)** water-soluble carbohydrate storages (mean ± SE) between the fencing and grazing conditions in three alpine grasslands located at site A–C, respectively. Different letters indicated significant difference for nutrition storages between the fencing and grazing conditions at *p* < 0.05 level.

**Figure 3:**
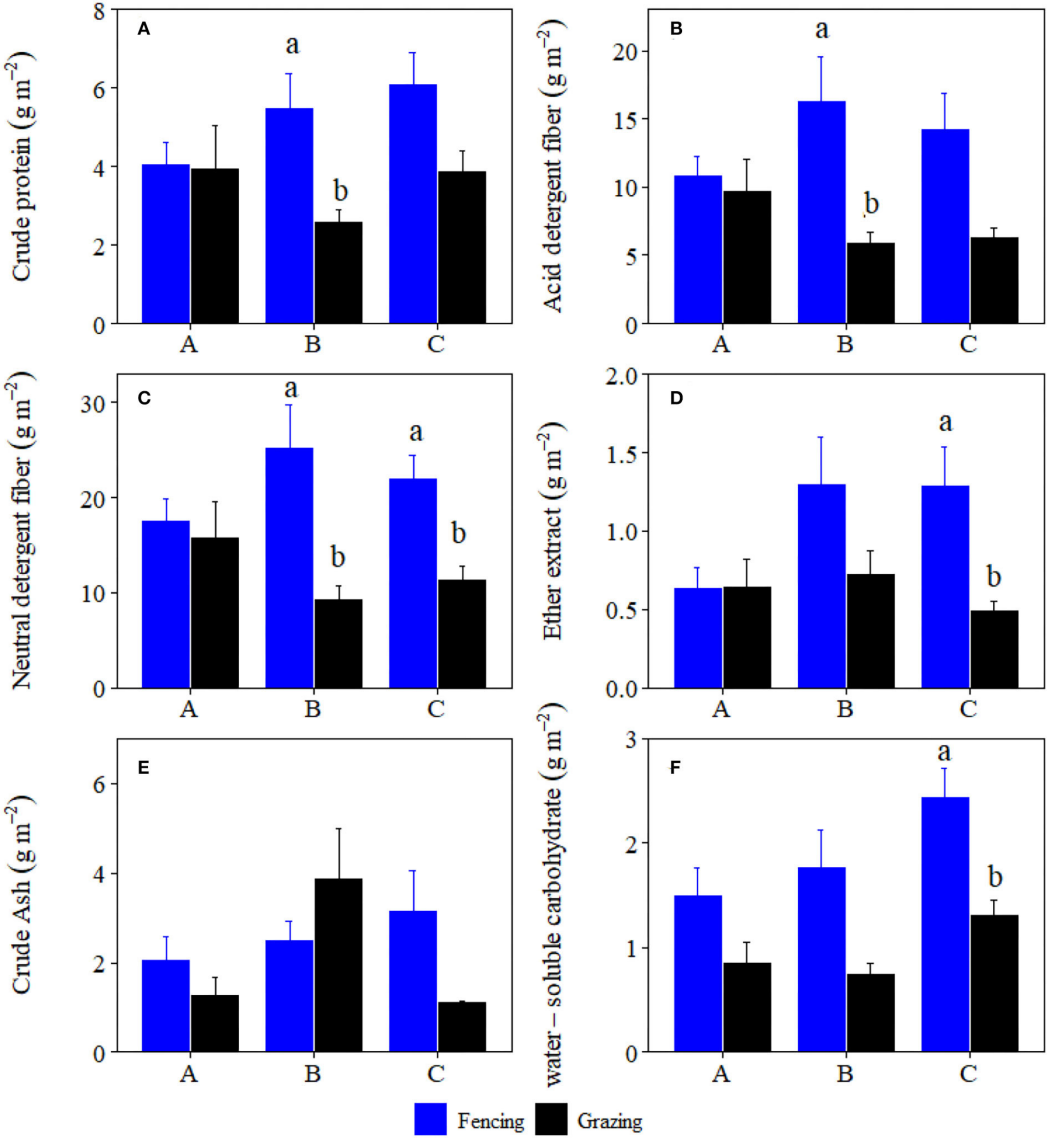
Comparisons of high-quality forage **(A)** crude protein, **(B)** acid detergent fiber, **(C)** neutral detergent fiber, **(D)** ether extract, **(E)** crude ash, and **(F)** water-soluble carbohydrate storages (mean ± SE) between the fencing and grazing conditions in three alpine grasslands located at site A–C, respectively.

**Figure 4:**
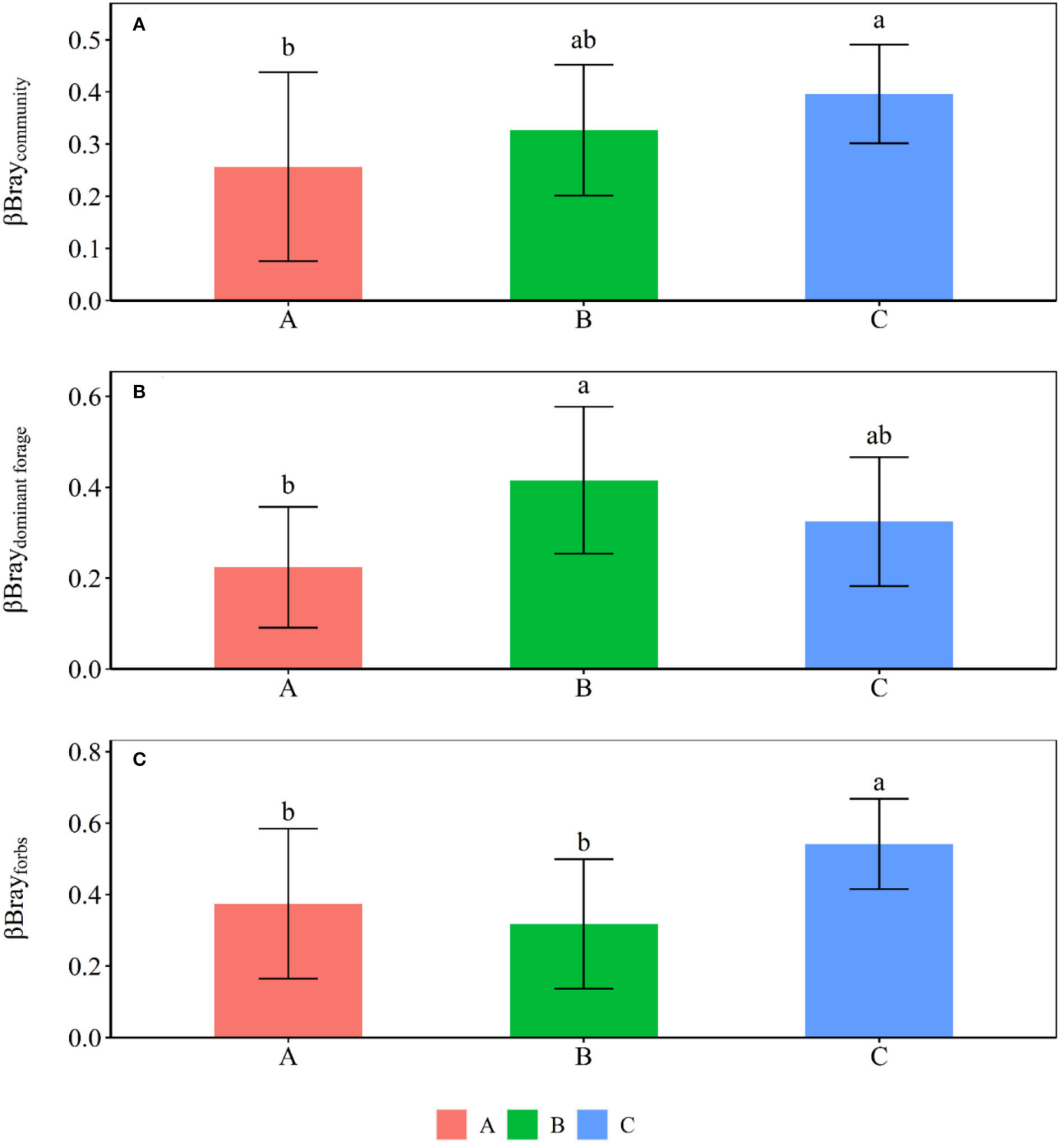
Comparisons of βBray (mean ± SD) between the fencing and grazing conditions at the three sites for **(A)** plant community, **(B)** high-quality forage, and **(C)** forbs, respectively. Different letters indicate significant difference at *p* < 0.05 level.

Grazing significantly reduced crude protein storage, acid detergent fiber storage, neutral detergent fiber storage, crude ash storage, ether extract storage, and water-soluble carbohydrates storage of plant community by 46.61, 62.47, 55.96, 64.94, 60.34, and 52.68%, and forbs by 62.33, 77.50, 73.69, 65.05, 57.75, and 62.44% at site C, respectively (Figures 1, 2). Grazing significantly reduced crude protein storage and acid detergent fiber storage of high-quality forage at site B by 53.29 and 63.82%, respectively (Figure 3). By contrast, grazing significantly reduced ether extract storage and water-soluble carbohydrates storage of high-quality forage at site C by 62.15 and 46.32%, respectively (Figure 3). Moreover, grazing significantly reduced neutral detergent fiber storage of high-quality forage at sites B and C by 63.26 and 48.14%, respectively (Figure 3). Compared to fencing, grazing significantly altered plant community and high-quality forage and forbs nutrient storages (i.e., the data matrix of CP, ADF, NDF, Ash, EE and WSC storages) at site C but not at sites A and B (Table 1). Grazing-induced changes in plant community and forbs nutrient storages at site A were significantly lower than those at site C (Figure 4). Grazing-induced changes in forbs nutrient storage at site B were significantly lower than that at site C (Figure 4). Grazing-induced changes of high-quality forage nutrient storage at site A were significantly lower than that at site B (Figure 4).

**Table 1:** Effects of free-grazing on nutrient storages of plant community, high-quality forage, and forbs based on the Adonis2 analysis for the three alpine grassland sites (A–C).

**Sites**	**Plant community**			**High-quality forage**			**Forbs**		
	**CV%**	***F***	***p***	**CV%**	***F***	***p***	**CV%**	***F***	***p***
A	70.53	0.95	0.169	59.31	0.57	0.508	56.14	1.02	0.254
B	38.42	2.57	0.137	38.89	7.97	0.059	57.12	0.62	0.478
C	23.87	31.76	0.030	43.68	11.90	0.030	23.31	24.03	0.029

### Impacts of grazing on the elevation distributions of crude protein storage, acid detergent fiber storage, neutral detergent fiber storage, crude ash storage, ether extract storage, water-soluble carbohydrates storage, and nutrient storages

**Figure 5:**
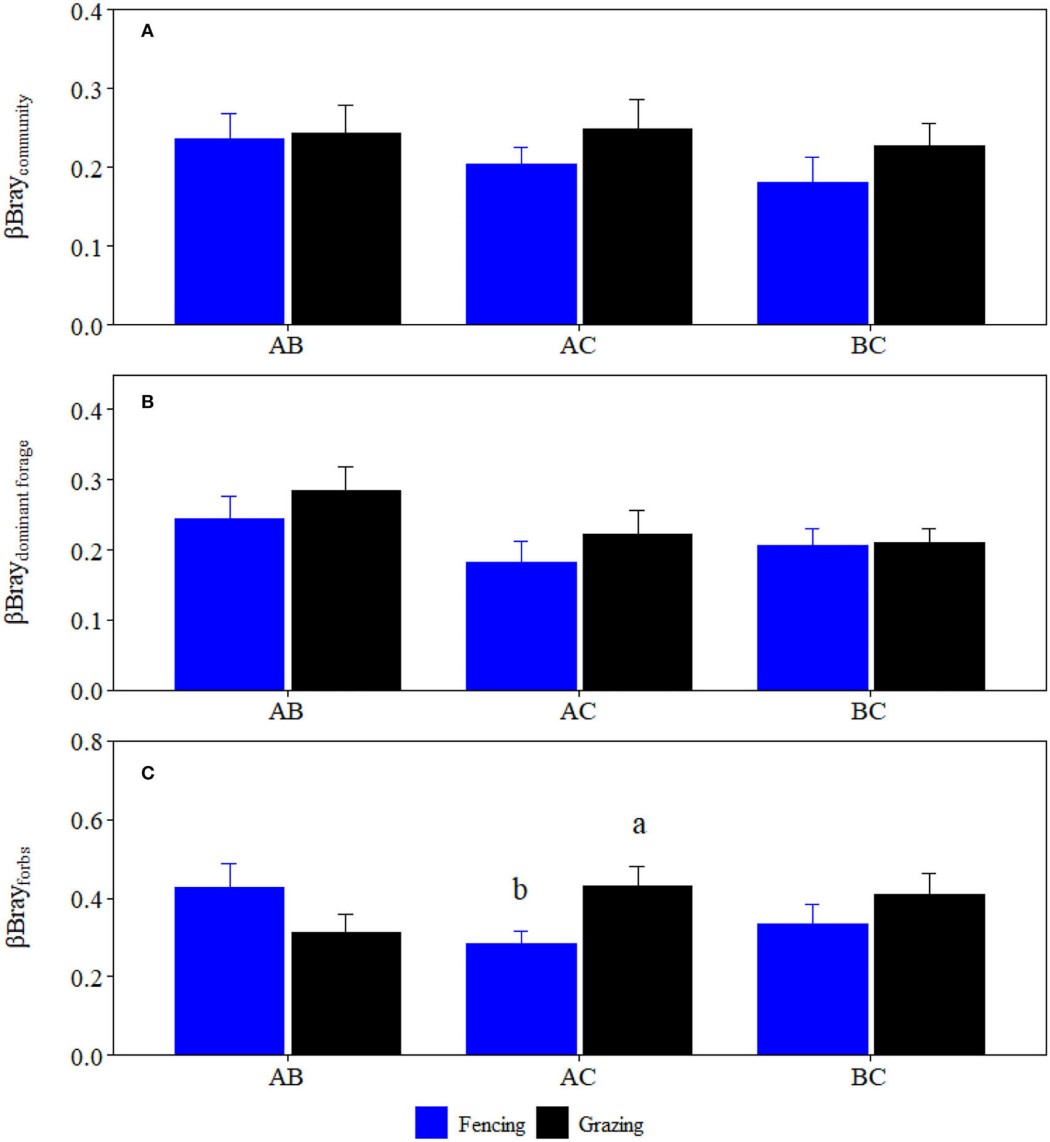
The βBray values (mean ± SE) between any two of the three sites for **(A)** plant community, **(B)** high-quality forage, and **(C)** forbs under fencing and grazing conditions, respectively. Different letters indicated significant difference at *p* < 0.05 level. AB, AC, and BC indicate the βBray between sites A and B, between sites A and C, and between sites B and C, respectively.

There were no significant differences of plant community crude protein storage, acid detergent fiber storage, neutral detergent fiber storage, crude ash storage, ether extract storage. and water-soluble carbohydrates storage among sites A, B, and C (Supplementary Figure S1). There were no significant differences of high-quality forage crude ash storage and water-soluble carbohydrates storage among site A, B, and C under fencing conditions (Supplementary Figure S2). By contrast, the high-quality forage crude ash storage at site B was significantly greater than that at sites A and C (Supplementary Figure S2). The high-quality forage water-soluble carbohydrates storage at site B was lower than that at site C (Supplementary Figure S2). Although there were no significant differences of forbs acid detergent fiber storage, ether extract storage, or crude ash storage among site A, B, and C under grazing conditions, the forbs acid detergent fiber storage, ether extract storage, and crude ash storage at site A were significantly greater than those at site B under fencing conditions (Supplementary Figure S3). Compared to fencing, grazing significantly increased the difference of forbs nutrient storages between sites A and C (Figure 5).

### Relationships of crude protein storage, acid detergent fiber storage, neutral detergent fiber storage, crude ash storage, ether extract storage, water-soluble carbohydrates storage, and nutrient storages, with environmental variables

The AP was negatively correlated with plant community crude ash storage, high-quality forage crude protein storage, crude ash storage, and nutrient storage, and forbs crude ash storage (Table 2). The ${\text{NH}}_{4}^{+}$-N was positively correlated with plant community water-soluble carbohydrates storage (Table 2). The C:N was positively correlated with high-quality forage water-soluble carbohydrates storage (Table 2). Available N:P was negatively correlated with high-quality forage acid detergent fiber storage, neutral detergent fiber storage, and nutrient storage (Table 2). The NH_**4**_^+^-N:NO_**3**_^−^-N was negatively correlated with forbs crude ash storage, ether extract storage, and crude ash storage (Table 2). The SR was positively correlated with plant community acid detergent fiber storage, neutral detergent fiber storage, and nutrient storage, and forbs nutrient storage (Table 3). The Shannon and Simpson indexes were positively correlated with plant community crude ash storage and ether extract storage (Table 3). The PD was significantly correlated with plant community acid detergent fiber storage, neutral detergent fiber storage, ether extract storage, and nutrient storages, high-quality forage crude protein storage, water-soluble carbohydrates storage, and forbs acid detergent fiber storage (Table 3). The βBray was positively correlated with plant community crude ash storage and high-quality forage water-soluble carbohydrates storage (Table 3). The βMNTD was significantly correlated with plant community crude ash storage, high-quality forage crude protein storage, neutral detergent fiber storage, ether extract storage, crude ash storage, water-soluble carbohydrates storage, and nutrient storages (Table 3).

**Table 2:** Correlation analysis between forage nutrient storages of plant community, high-quality forage or forbs, and soil variables.

	**Variables**	**SOC**	**TN**	**TP**	**${\text{NH}}_{4}^{+}$-N**	**${\text{NO}}_{3}^{-}$-N**	**AP**	**pH**	**C:N**	**C:P**	**N:P**	**Available N:P**	**${\text{NH}}_{4}^{+}$-N:${\text{NO}}_{3}^{-}$-N**
Plant community	CP storage	−0.06	−0.05	0.04	0.02	−0.08	−0.03	−0.02	−0.03	−0.05	−0.04	−0.04	−0.08
	ADF storage	−0.01	−0.01	0.01	0.02	0.00	−0.10	−0.01	−0.01	−0.01	0.01	−0.09	−0.02
	NDF storage	−0.03	−0.02	−0.01	−0.01	0.01	−0.10	−0.02	−0.02	−0.02	−0.01	−0.09	−0.05
	EE storage	0.02	0.02	0.04	0.00	−0.03	−0.10	0.02	0.01	0.00	0.00	0.02	−0.11
	Ash storage	0.02	0.05	−0.03	0.00	0.03	−0.15^*^	0.01	−0.03	−0.01	0.01	0.03	−0.09
	WSC storage	0.01	0.01	0.11	0.13^*^	−0.04	0.07	0.05	0.04	0.00	−0.01	0.03	−0.01
	Nutrient storages	−0.02	−0.02	0.00	0.00	−0.01	−0.11	−0.02	−0.03	−0.03	−0.01	−0.07	−0.07
High-quality forage	CP storage	−0.03	−0.04	0.00	−0.01	−0.11	−0.12^*^	−0.02	0.00	−0.01	0.01	−0.11	−0.06
	ADF storage	−0.07	−0.07	−0.01	−0.03	−0.02	−0.08	−0.09	−0.05	−0.05	−0.04	−0.13^*^	−0.03
	NDF storage	−0.10	−0.10	−0.03	−0.08	−0.06	−0.10	−0.11	−0.08	−0.09	−0.08	−0.16^**^	−0.08
	EE storage	0.01	−0.02	−0.01	−0.01	−0.09	−0.06	0.03	0.05	0.04	0.02	−0.03	−0.05
	Ash storage	−0.05	−0.05	0.07	−0.01	0.00	−0.13^*^	−0.01	−0.06	0.00	0.02	0.02	−0.07
	WSC storage	0.09	0.08	0.11	0.10	−0.06	−0.04	0.08	0.12^*^	0.10	0.08	−0.06	0.07
	Nutrient storages	−0.08	−0.08	−0.01	−0.06	−0.06	−0.12^*^	−0.09	−0.06	−0.06	−0.05	−0.15^*^	−0.07
Forbs	CP storage	−0.04	−0.02	0.04	0.03	−0.06	0.04	−0.02	−0.06	−0.08	−0.08	0.09	−0.12^*^
	ADF storage	0.00	0.00	0.04	0.08	−0.05	0.00	0.01	−0.02	−0.03	−0.03	0.00	−0.03
	NDF storage	0.03	0.05	0.01	0.08	0.01	−0.02	0.05	−0.01	0.00	0.00	0.07	−0.08
	EE storage	−0.02	0.00	0.08	0.02	−0.03	−0.05	0.00	−0.06	−0.07	−0.05	0.09	−0.17^**^
	Ash storage	0.00	0.04	−0.02	−0.04	0.02	−0.15^*^	0.00	−0.05	−0.02	0.00	−0.01	−0.13^*^
	WSC storage	−0.02	0.00	0.05	0.02	−0.03	0.03	0.01	−0.02	−0.05	−0.05	0.06	−0.07
	Nutrient storages	0.00	0.03	0.03	0.05	−0.02	−0.04	0.02	−0.03	−0.03	−0.02	0.05	−0.10

** and

* indicated significant correlation at *p* < 0.01 and *p* < 0.05, respectively.

**Table 3:** Correlation analysis between forage nutrient storages of plant community, high-quality forage or forbs, and biotic factors.

	**Variables**	**SR**	**Shannon**	**Simpson**	**Pielou**	**PD**	**MNTD**	**βBray**	**βMNTD**
Plant community	CP storage	0.11	−0.09	−0.08	−0.01	0.11	0.03	−0.02	−0.02
	ADF storage	0.22^***^	−0.02	−0.02	0.01	0.22^***^	0.04	−0.01	0.05
	NDF storage	0.19^**^	−0.05	−0.06	−0.03	0.18^**^	0.03	−0.02	0.05
	EE storage	0.12	0.17^**^	0.12^*^	0.03	0.12^*^	0.04	0.04	0.06
	Ash storage	−0.01	0.25^***^	0.20^**^	0.04	0.02	0.06	0.15^*^	0.19^**^
	WSC storage	0.11	−0.06	−0.05	0.04	0.10	0.08	0.07	0.02
	Nutrient storages	0.17^**^	0.00	−0.02	−0.01	0.17^**^	0.04	0.02	0.06
High-quality forage	CP storage	−0.09	0.00	−0.01	0.03	−0.14^*^	−0.03	0.03	−0.18^**^
	ADF storage	0.02	−0.05	−0.05	−0.02	−0.01	−0.08	−0.08	−0.10
	NDF storage	0.01	−0.09	−0.08	−0.06	−0.04	−0.08	−0.08	−0.12^*^
	EE storage	0.05	−0.03	−0.01	−0.01	0.00	0.02	−0.03	−0.18^**^
	Ash storage	−0.07	0.07	0.07	0.07	−0.03	−0.05	0.03	−0.12^*^
	WSC storage	−0.11	0.01	0.01	0.05	−0.12^*^	0.07	0.18^**^	−0.16^**^
	Nutrient storages	−0.01	−0.06	−0.06	−0.03	−0.05	−0.07	−0.05	−0.15^*^
Forbs	CP storage	0.03	0.00	0.01	−0.01	0.00	−0.03	−0.02	0.02
	ADF storage	0.17^**^	0.10	0.09	0.03	0.15^*^	0.04	0.02	0.09
	NDF storage	0.08	0.01	0.00	−0.03	0.08	−0.05	0.04	0.10
	EE storage	0.01	0.00	0.02	0.00	0.00	−0.07	0.01	0.04
	Ash storage	−0.07	−0.09	−0.07	−0.03	−0.04	−0.08	0.07	0.05
	WSC storage	0.12	0.08	0.09	0.07	0.09	0.06	0.01	0.04
	Nutrient storages	0.06	0.00	0.00	−0.02	0.06	−0.04	0.03	0.08

*** ,

** and

* indicated significant correlation at *p* < 0.001, *p* < 0.01 and *p* < 0.05, respectively.

**Figure 6:**
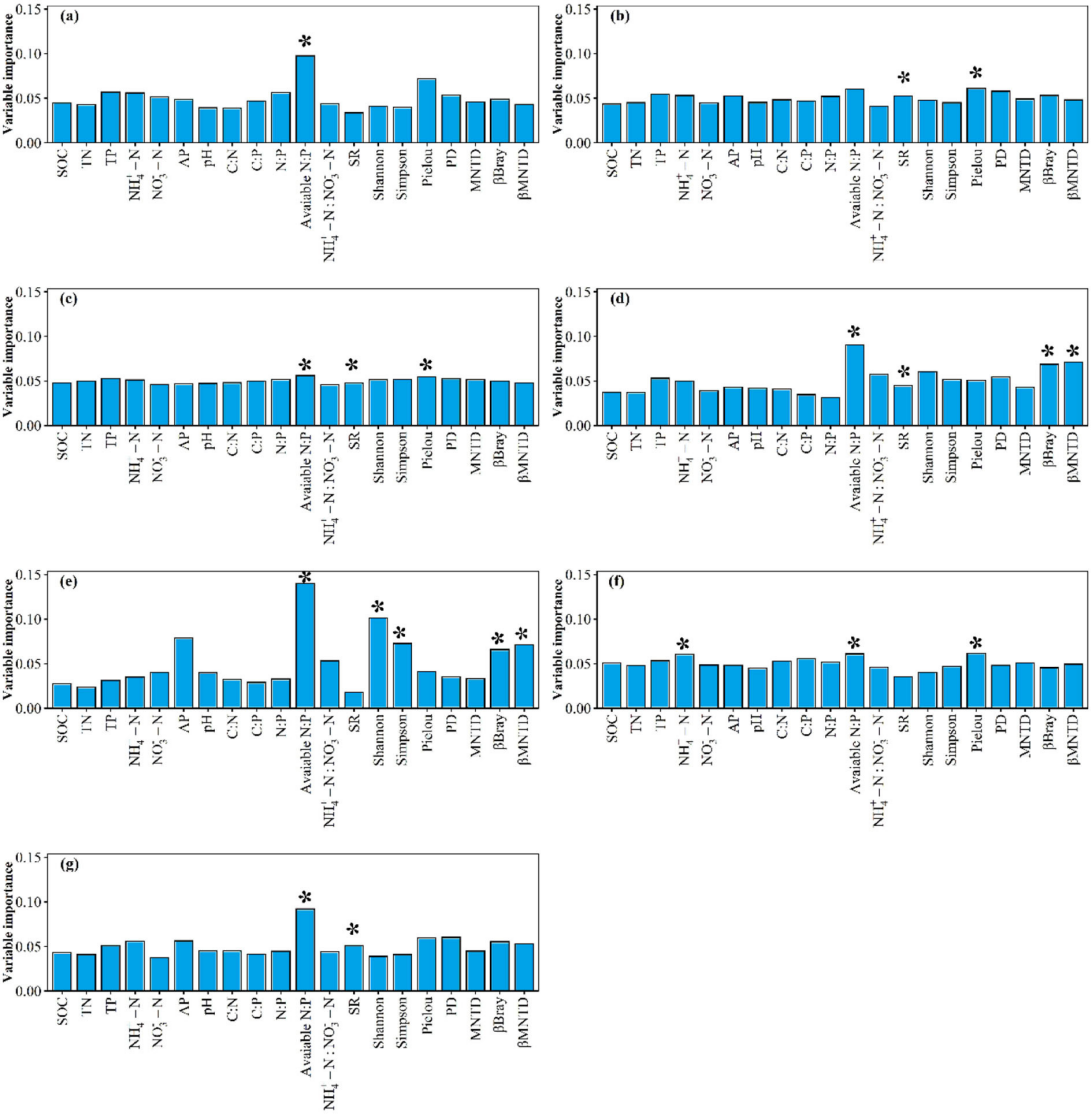
The relative contributions of α- and β-diversity of plant community and soil variables for plant community **(a)** crude protein storage, **(b)** acid detergent fiber storage, **(c)** neutral detergent fiber storage, **(d)** ether extract storage, **(e)** crude ash storage, **(f)** water-soluble carbohydrates storage, and **(g)** nutrient storages, respectively. * indicates *p* < 0.05.

**Figure 7:**
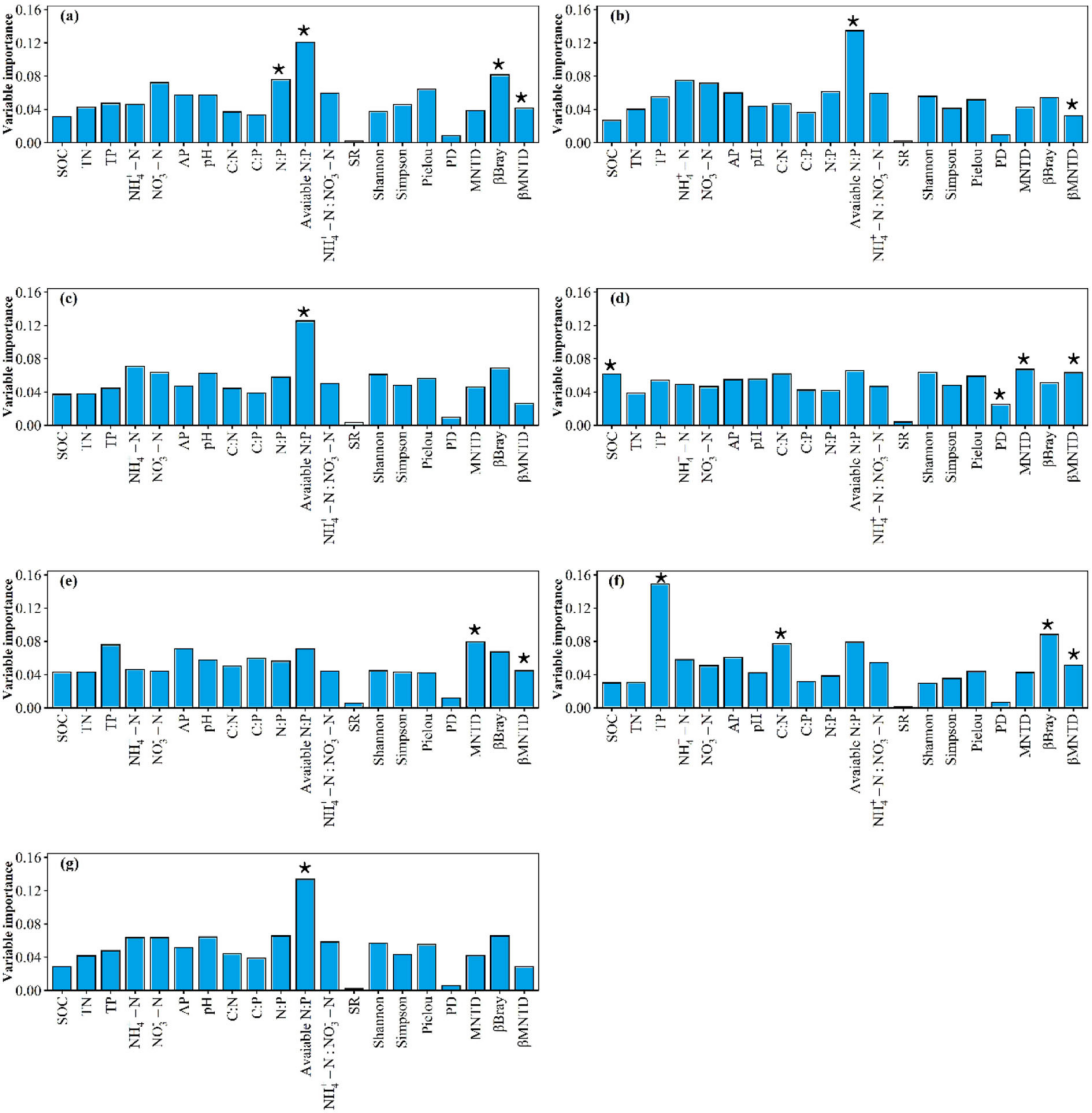
The relative contributions of α- and β-diversity of high-quality forage and soil variables for high-quality forage **(a)** crude protein storage, **(b)** acid detergent fiber storage, **(c)** neutral detergent fiber storage, **(d)** ether extract storage, **(e)** crude ash storage, **(f)** water-soluble carbohydrates storage, and **(g)** nutrient storages, respectively. * indicates *p* < 0.05.

**Figure 8:**
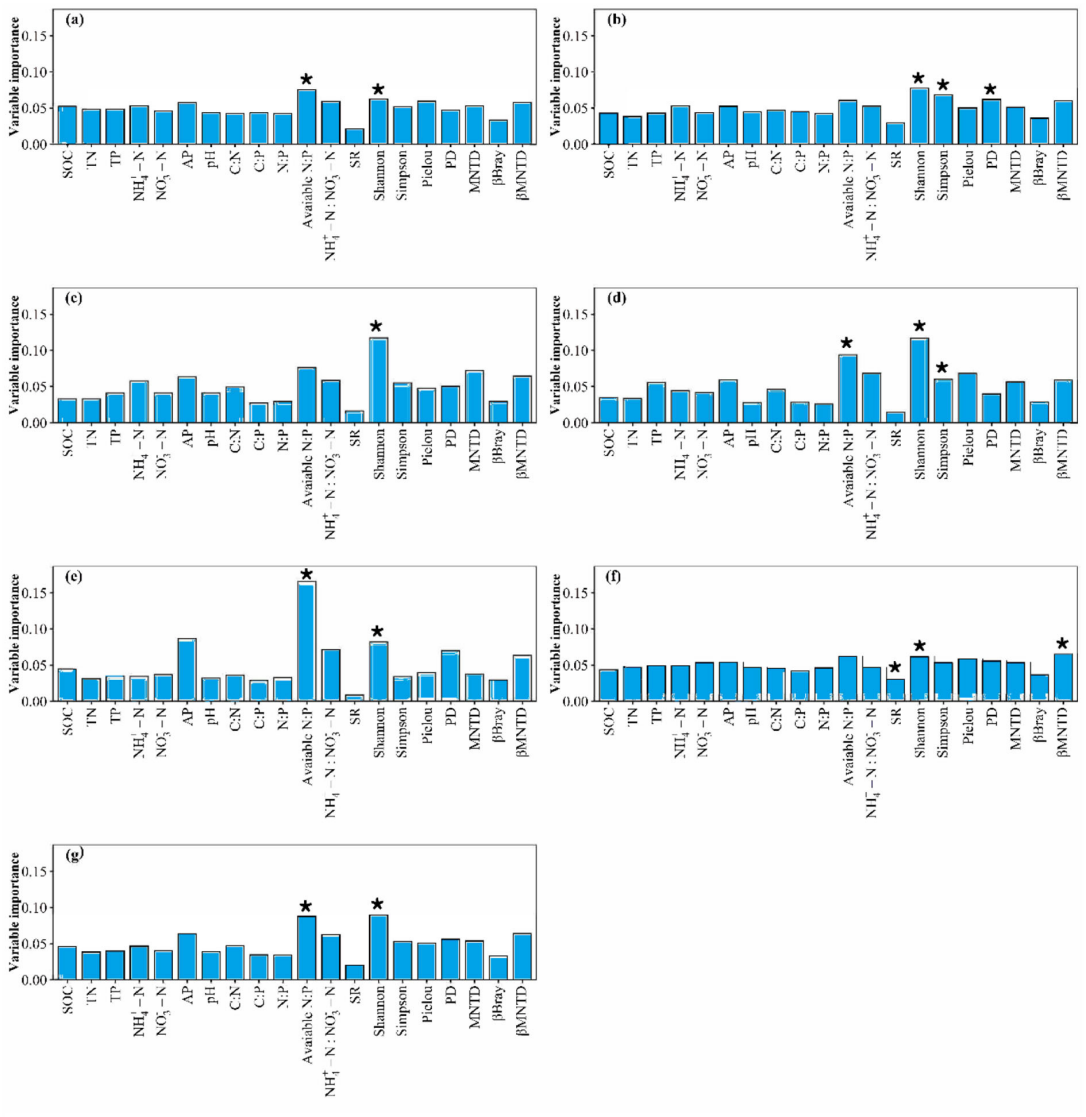
The relative contributions of α- and β-diversity of forbs and soil variables for forbs **(a)** crude protein storage, **(b)** acid detergent fiber storage, **(c)** neutral detergent fiber storage, **(d)** ether extract storage, **(e)** crude ash storage, **(f)** water-soluble carbohydrates storage, and **(g)** nutrient storages, respectively. * indicates *p* < 0.05.

Availability N:P predominated the variation of plant community crude protein storage (Figure 6a). Plant community SR and Pielou predominated the variation of plant community acid detergent fiber storage (Figure 6b). Availability N:P, plant community SR and Pielou predominated the variation of plant community neutral detergent fiber storage (Figure 6c). Availability N:P, plant community SR, βBray and βMNTD predominated the variation of plant community ether extract storage (Figure 6d). Availability N:P, plant community Shannon, Simpson, βBray and βMNTD predominated the variation of plant community crude ash storage (Figure 6e). Availability N:P, NH_4_+-N and plant community Pielou predominated the variation of plant community water-soluble carbohydrates storage (Figure 6f). Availability N:P and plant community SR predominated the variation of plant community nutrient storages (Figure 6g). Availability N:P, N:P, high-quality forage βBray and βMNTD predominated the variation of high-quality forage crude protein storage (Figure 7a). Availability N:P, and high-quality forage βMNTD predominated the variation of high-quality forage acid detergent fiber storage (Figure 7b). Availability N:P predominated the variation of high-quality forage neutral detergent fiber storage (Figure 7c). The SOC, high-quality forage PD, MNTD, and βMNTD predominated the variation of high-quality forage ether extract storage (Figure 7d). High-quality forage MNTD and βMNTD predominated the variation of high-quality forage crude ash storage (Figure 7e). The TP, C:N, high-quality forage βBray and βMNTD predominated the variation of high-quality forage water-soluble carbohydrates storage (Figure 7f). Availability N:P predominated the variation of high-quality forage nutrient storages (Figure 7g). Availability N:P, and forbs Shannon predominated the variation of forbs crude protein storage, crude ash storage, and nutrient storages (Figures 8a,e,g). Forbs Shannon, Simpson, and PD predominated the variation of forbs acid detergent fiber storage (Figure 8b). Forbs Shannon predominated the variation of forbs neutral detergent fiber storage (Figure 8c). Availability N:P, forbs Shannon and Simpson predominated the variation of forbs ether extract storage (Figure 8d). Forbs SR, Shannon and βMNTD predominated the variation of forbs water-soluble carbohydrates storage (Figure 8f).

## Discussion

Consistent with our hypothesis (H1), compared to cold-season grazing, warm-season grazing can have greater negative effects on forage nutrient storages, mainly on nutrient storages of high-quality forage (in detail, mainly on CP, ADF and NDF storages). This finding was similar with some earlier studies (Sun et al., 2021; Zhang and Fu, 2021) and may be attributed to at least one or more of the following mechanisms. First, available N:P was the leading predominant variable controlling the variations of nutrient storages, CP, ADF and NDF storages of high-quality forage, and negatively correlated with nutrient storages, CP, ADF and NDF storages of high-quality forage. By contrast, cold-season grazing caused a greater magnitude change in available N:P compared to warm-season grazing (Fu et al., 2021). Second, βMNTD was another predominant variable controlling the variations of CP and ADF storages of high-quality forage, and negatively correlated with CP and ADF storages of high-quality forage. By contrast, the βMNTD of high-quality forage between cold-season and ungrazing conditions was greater than that between warm-season and ungrazing conditions (Fu et al., 2021). This phenomenon cautioned that cold-season grazing did not always have greater negative effects on alpine grassland ecosystems than warm-season grazing, at least in the Northern Tibetan Plateau. Moreover, the impacts of both cold-season grazing and warm-season grazing on grassland ecosystems should receive the same attention in alpine regions.

Our findings implied that the impacts of warm-season grazing on forage nutrient storages can vary within alpine grassland ecosystems. This finding was similar to some earlier studies (Xiong et al., 2016), and may be due to at least one of the subsequent mechanisms. First, different alpine grassland ecosystems can generally have different species diversity and community composition (Wu et al., 2014a, 2015), and different species can have different nutrient quality and plant aboveground biomass production (Sun et al., 2020; Fu et al., 2021). External disturbance may result in a new community assembly (Sun et al., 2021; Wang et al., 2021a,b), and in turn may lead to new forage nutrient quality and storages in grassland ecosystems. Moreover, the impacts of grazing on plant community assembly can vary with alpine grassland ecosystems (Sun et al., 2021; Zhang and Fu, 2021). Second, soil carbon, nitrogen, and phosphorous availability can also affect forage nutrient quality and biomass accumulation to some extent (Fu and Shen, 2016; Wang et al., 2021a). For example, soil ${\text{NH}}_{4}^{+}$-N and C:N was a predominant variable for WSC storages of plant community and high-quality forage, respectively, in this study. Summer grazing significantly altered soil ${\text{NH}}_{4}^{+}$-N and C:N in the alpine meadow but not the alpine steppe meadow (Fu et al., 2021). Third, soil microbial community can be closely correlated with plant community, and may influence forage nutrient storages in alpine grassland ecosystems (Zhang et al., 2020; Zhang and Fu, 2021). The impacts of grazing on the soil microbial community can vary with alpine grassland ecosystems. This phenomenon further implied that alpine grassland ecosystems should adopt different grazing management strategies.

Consistent with our hypothesis (H2), plant species and phylogenetic diversity can have different correlations with forage nutrient storages, and α- and β-diversity can also have different correlations with forage nutrient storages. This finding might be due to at least one of the subsequent mechanisms. First, although both plant species and phylogenetic diversity can have some connection, no two share exactly the same characteristics of plant community (Fu et al., 2021; Sun et al., 2021). Meanwhile, the correlations between forage nutrient quality and plant species diversity can be different from those between forage nutrient quality and plant phylogenetic diversity (Fu et al., 2021). Second, although both α- and β-diversity can have a certain relationship, no two share exactly the same characteristics of biodiversity (Zhang et al., 2021; Zhong and Fu, 2022). Moreover, forage nutrient quality can have different correlations with plant α- and β-diversity (Fu et al., 2021). This phenomenon indicates that a combination of plant species and phylogenetic α- and β-diversity can be better in predicting the correlations between forage nutrient storages and plant diversity.

Consistent with our hypothesis (H3), grazing can reconstruct the elevational pattern of forage nutrient storages in alpine grassland ecosystems (mainly reflected in forbs nutrient storage between sites A and C), which was in agreement with earlier studies (Zhang and Fu, 2022). This finding might be due to at least one of the subsequent mechanisms. First, SOC, ${\text{NH}}_{4}^{+}$-N, ${\text{NO}}_{3}^{-}$-N, pH, C:N, N:P and ${\text{NH}}_{4}^{+}$-N:${\text{NO}}_{3}^{-}$-N were not the predominant factors controlling the variations of forbs nutrient storage. However, grazing significantly altered the differences of SOC, ${\text{NH}}_{4}^{+}$-N, ${\text{NO}}_{3}^{-}$-N, pH, C:N, N:P and ${\text{NH}}_{4}^{+}$-N:${\text{NO}}_{3}^{-}$-N between site A and C (Supplementary Figures S4, S5). Secondly, availability N:P was one of the predominant factors controlling the variation of forbs nutrient storage, but grazing significantly reduced the difference of availability N:P between site A and C (Supplementary Figure S5). Thirdly, the forbs Shannon index was another predominant factor controlling the variation of forbs nutrient storage, but grazing did not significantly alter the difference of Shannon between site A and C (Supplementary Figure S6). Fourthly, although forbs SR and MNTD were not the predominant factors controlling the variation of forbs nutrient storage, grazing significantly altered the differences of SR and MNTD between site A and C (Supplementary Figure S6). Lastly, grazing did not significantly alter forbs βBray and βMNTD values between site A and C (Supplementary Figure S7). Meanwhile, forbs βBray and βMNTD values were not the predominant factors controlling the variations of forbs nutrient storage.

## Conclusions

Plant species and phylogenetic diversity had different correlations with forage nutrient storage, and plant α- and β-diversity also had different correlations with forage nutrient storage. This implied a high probability that a combination of plant species and phylogenetic α- and β-diversity had closer correlations with forage nutrient storage under grazing conditions. Cold-season free-grazing had a lower negative effect on nutrient storages of high-quality forage than warm-season free-grazing, which implied that cold-grazing did not always cause greater disturbances and degradations on alpine grassland ecosystems than warm-season free-grazing. Warm-season free-grazing had a greater impact on nutrient storages of forbs in alpine meadow than alpine steppe meadow, which implied that the impact of grazing on alpine grassland ecosystems varied with grassland types. Grazing may increase the differences of forage nutrient storages among elevations, which implied that human activities probably do and will continue to alter the spatial patterns of alpine grassland ecosystems. All of these observations further confirmed that the classification management of alpine grassland ecosystems is an important aspect of its adaptive management on the Tibetan Plateau.

## Data availability statement

The original contributions presented in the study are included in the article/Supplementary material, further inquiries can be directed to the corresponding author/s.

## Author contributions

XZ and GF: conceptualization, methodology, writing—original draft preparation, writing—review and editing, visualization, supervision, project administration, and funding acquisition. YT: software. XZ, GF, and O: validation. GF: formal analysis and investigation. YT: resources. XZ: data curation. All authors have read and agreed to the published version of the manuscript.

## Funding

This research was funded by Youth Innovation Promotion Association of Chinese Academy of Sciences [2020054], National Natural Science Foundation of China [31600432], Bingwei Outstanding Young Talents Program of Institute of Geographic Sciences and Natural Resources Research, Chinese Academy of Sciences [2018RC202], Science and Technology Project of Tibet Autonomous Region [XZ202101ZD0003N, XZ202202YD0009C, XZ202101ZD0007G, and XZ202201ZY0003N], and Construction of Fixed Observation and Experimental Station of First and Try Support System for Agricultural Green Development in Zhongba County.

## Conflict of interest

The authors declare that the research was conducted in the absence of any commercial or financial relationships that could be construed as a potential conflict of interest.

## Publisher's note

All claims expressed in this article are solely those of the authors and do not necessarily represent those of their affiliated organizations, or those of the publisher, the editors and the reviewers. Any product that may be evaluated in this article, or claim that may be made by its manufacturer, is not guaranteed or endorsed by the publisher.
