# Supplementary material for: Response of forage nutrient storages to grazing in alpine grasslands
Source: Front Plant Sci. 2022 Nov 1;13:991287. doi: 10.3389/fpls.2022.991287 (PMC9664390; doi:10.3389/fpls.2022.991287)
Supplement: Supplementary file 1 [file Presentation_1.pdf]

Response of forage nutrient storages to grazing in alpine grasslands

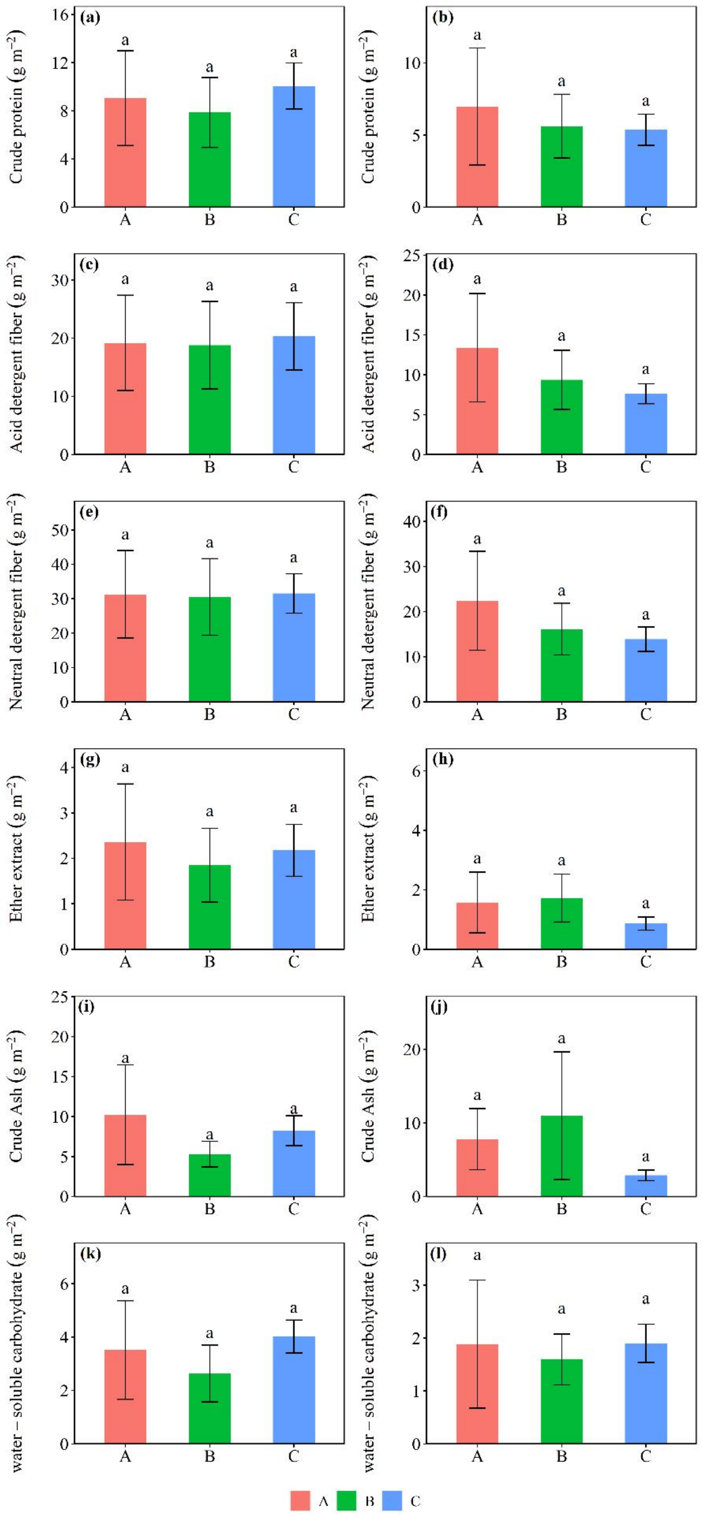

Figure S1 Comparison of plant community crude protein, acid detergent fiber, neutral detergent fiber, ether extract, crude ash and water-soluble carbohydrates pools (mean ± SD) among sites A, B and C under fencing (a, c, e, g, i, k) or grazing (b, d, f, h, j, l) conditions, respectively.

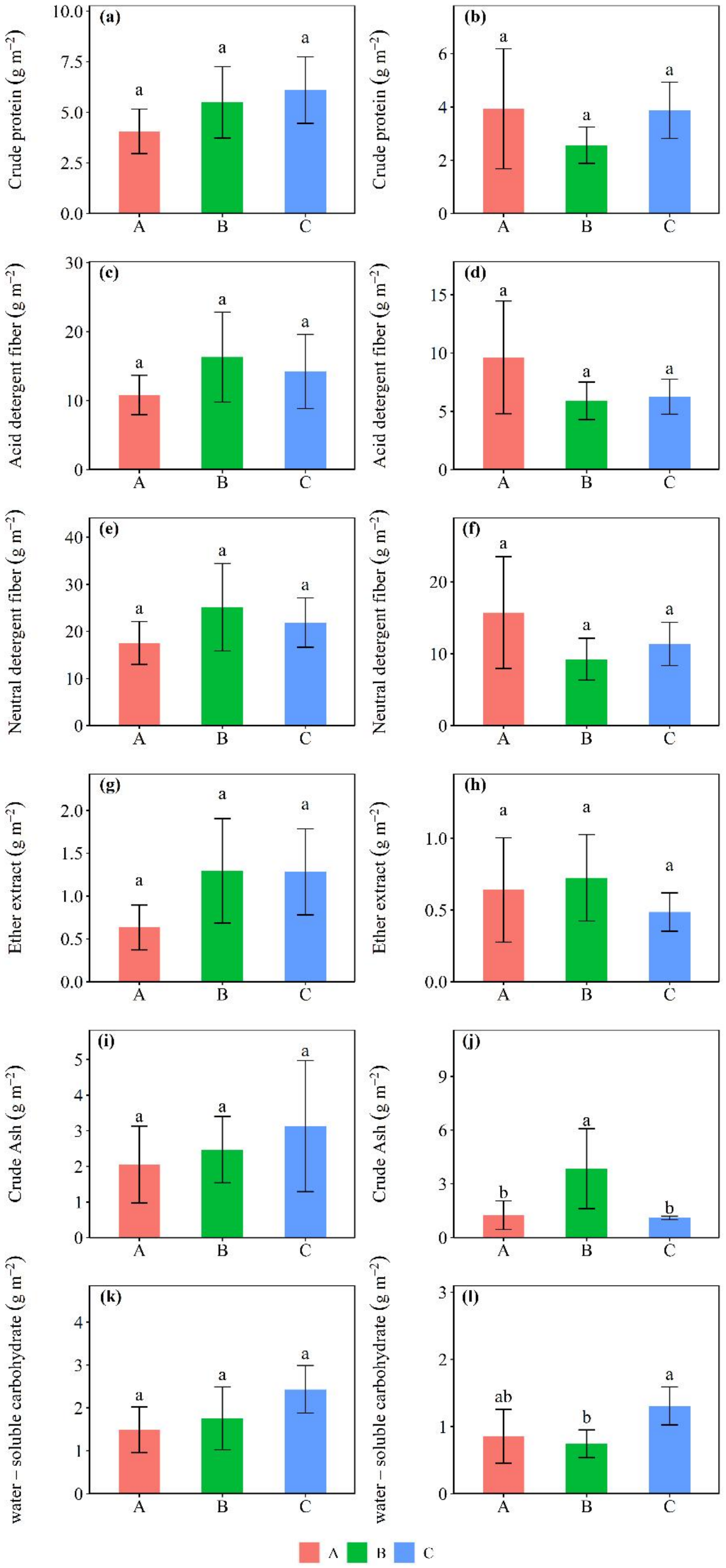

Figure S2 Comparison of high-quality forage crude protein, acid detergent fiber, neutral detergent fiber, ether extract, crude ash and water-soluble carbohydrates pools (mean ± SD) among sites A, B and C under fencing (a, c, e, g, i, k) or grazing (b, d, f, h, j, l) conditions, respectively.

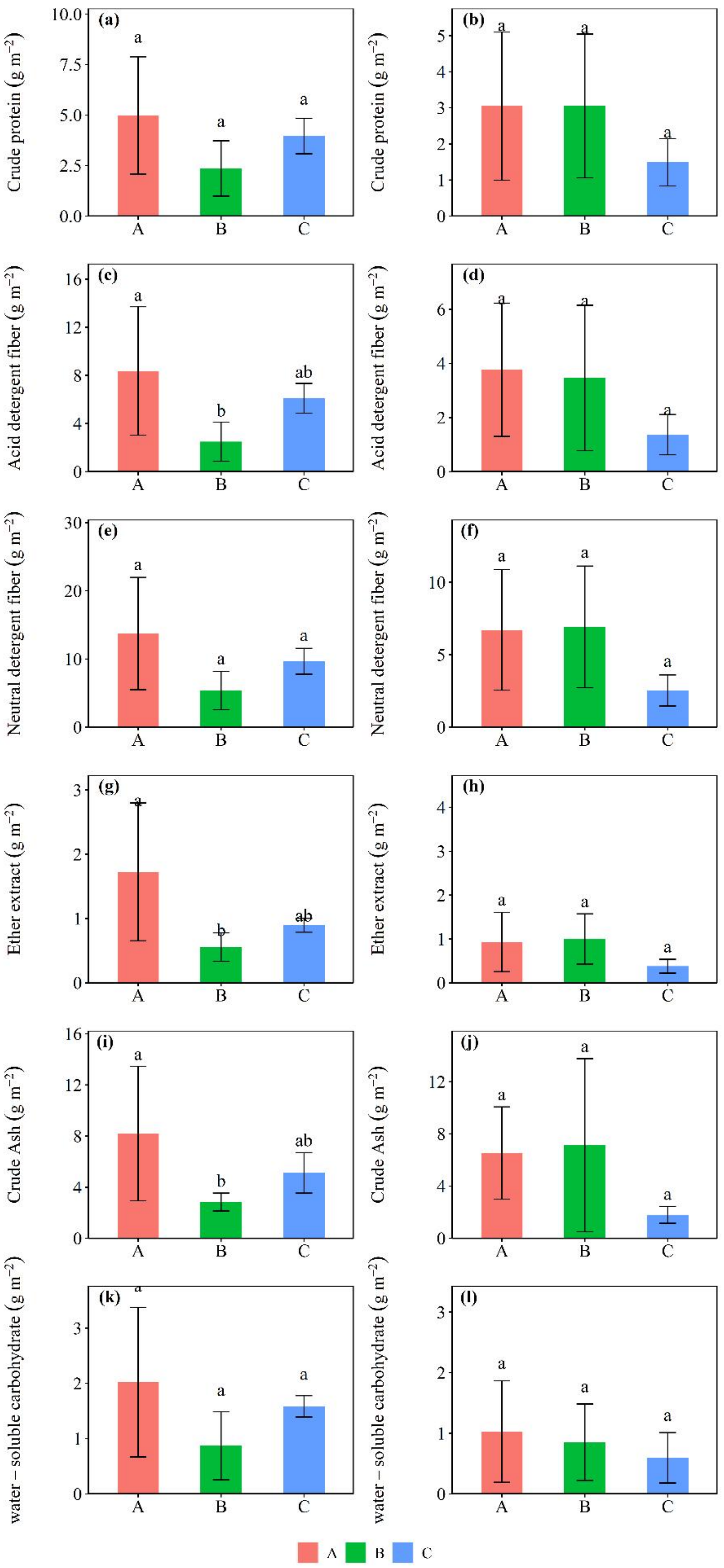

Figure S3 Comparison of forbs crude protein, acid detergent fiber, neutral detergent fiber, ether extract, crude ash and water-soluble carbohydrates pools (mean ± SD) among sites A, B and C under fencing (a, c, e, g, i, k) or grazing (b, d, f, h, j, l) conditions, respectively.

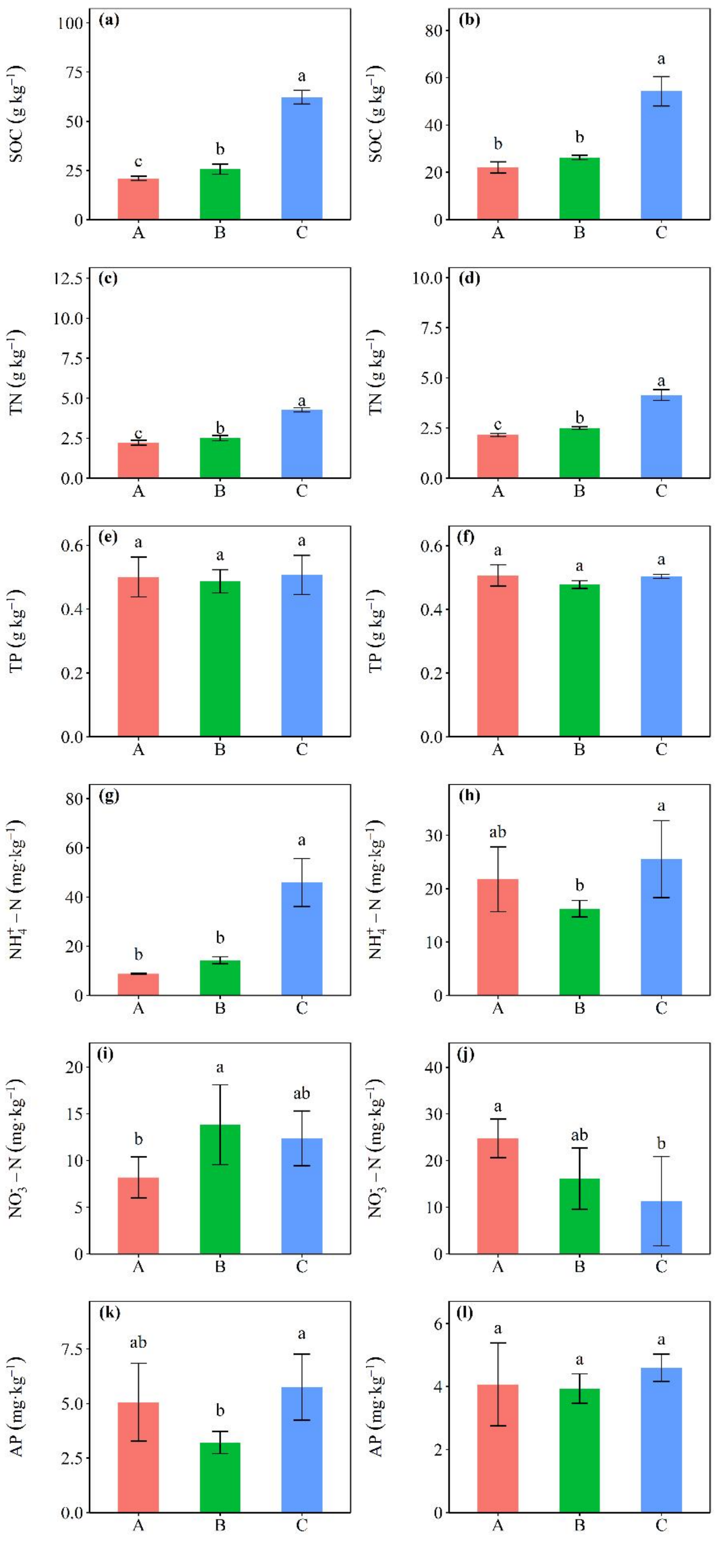

Figure S4 Comparison of soil organic carbon (SOC), total nitrogen (TN), total phosphorus (TP), ammonium nitrogen (NH_4^+-N), nitrate nitrogen (NO_3^--N) and available phosphorus (AP) concentration (mean ± SD) among sites A, B and C under fencing (a, c, e, g, i, k) or grazing (b, d, f, h, j, l) conditions, respectively.

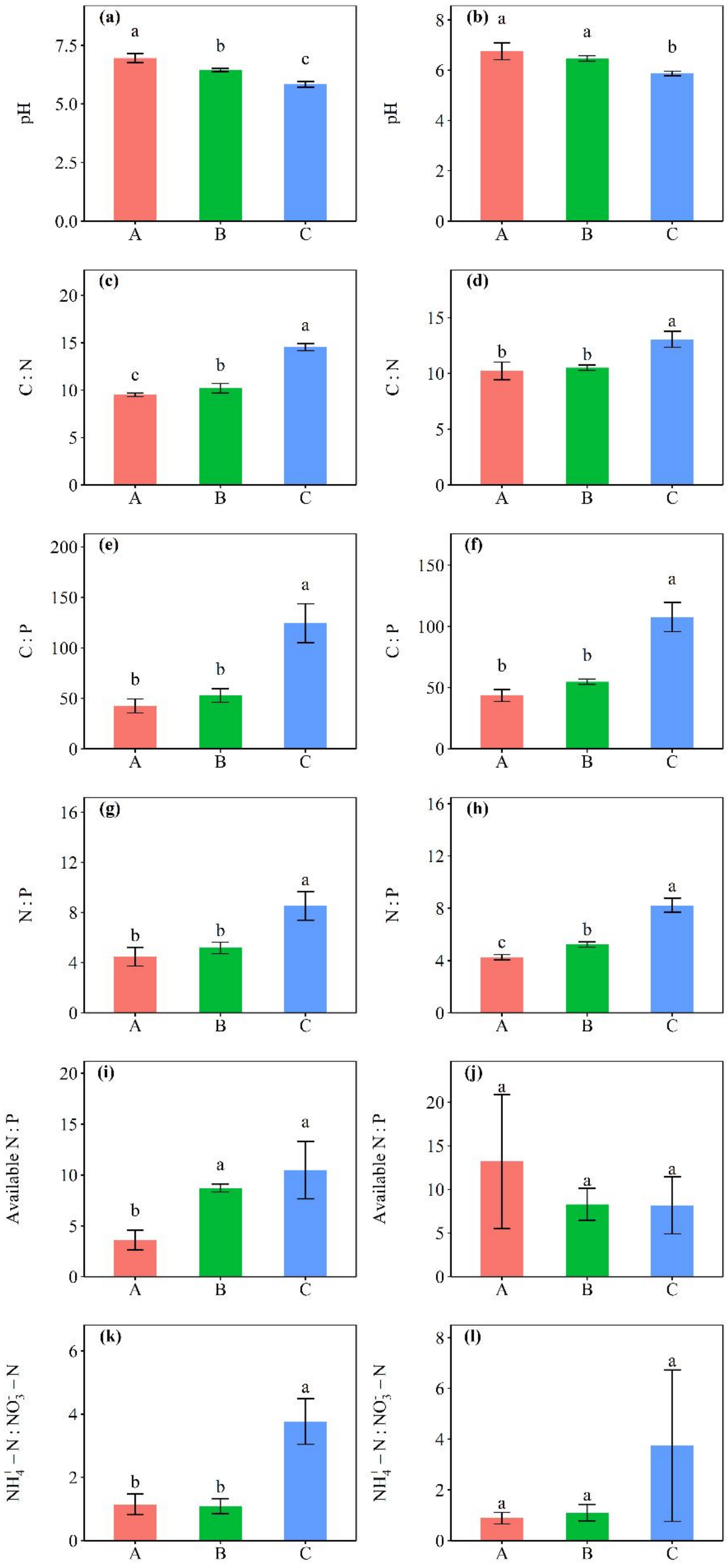

Figure S5 Comparison of pH, C:N, C:P, N:P, available N:P and NH_4^+-N:NO_3^--N (mean ± SD) among sites A, B and C under fencing (a, c, e, g, i, k) or grazing (b, d, f, h, j, l) conditions, respectively.

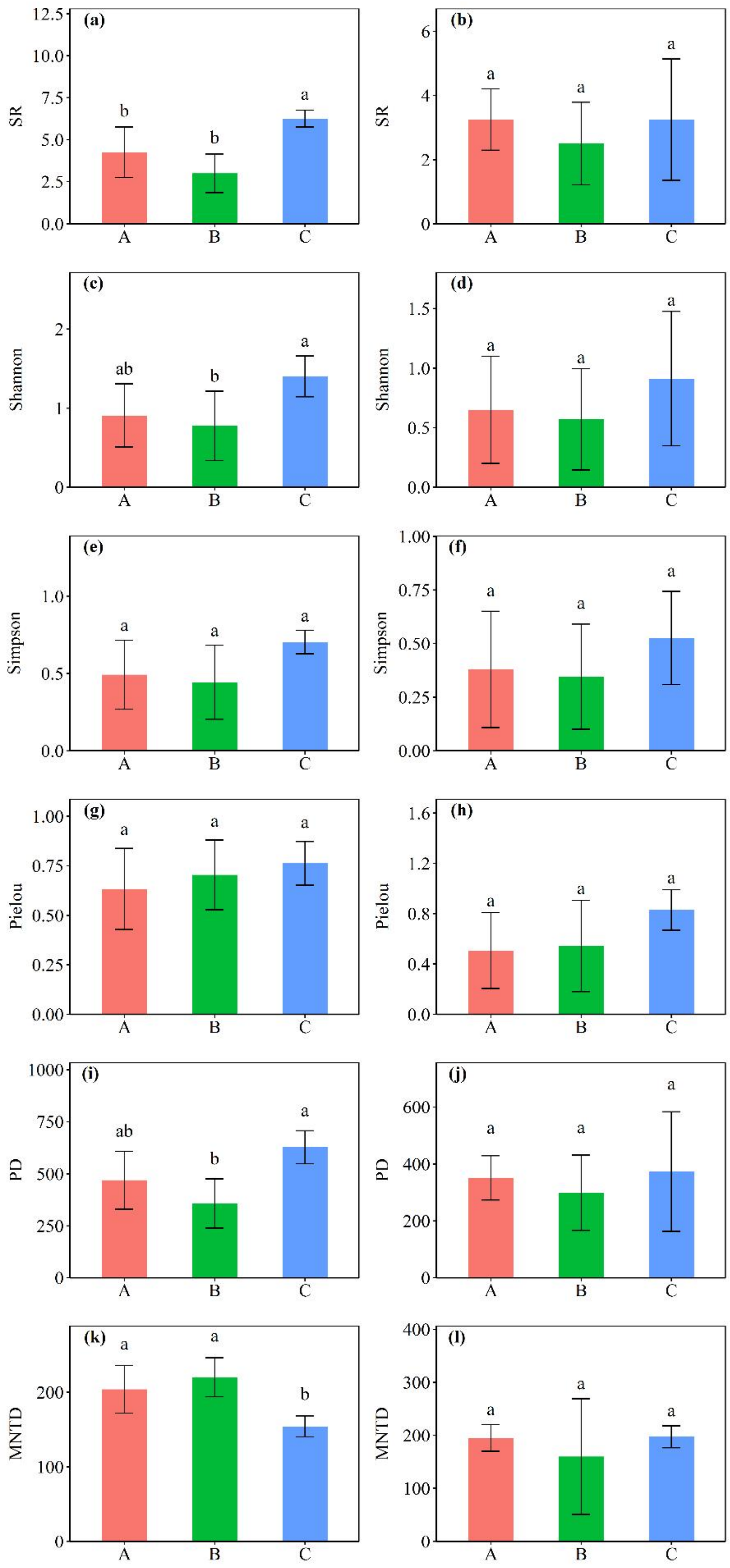

Figure S6 Comparison of species richness (SR), Shannon, Simpson, Pielou, PD and MNTD (mean ± SD) among sites A, B and C under fencing (a, c, e, g, i, k) or grazing (b, d, f, h, j, l) conditions, respectively.

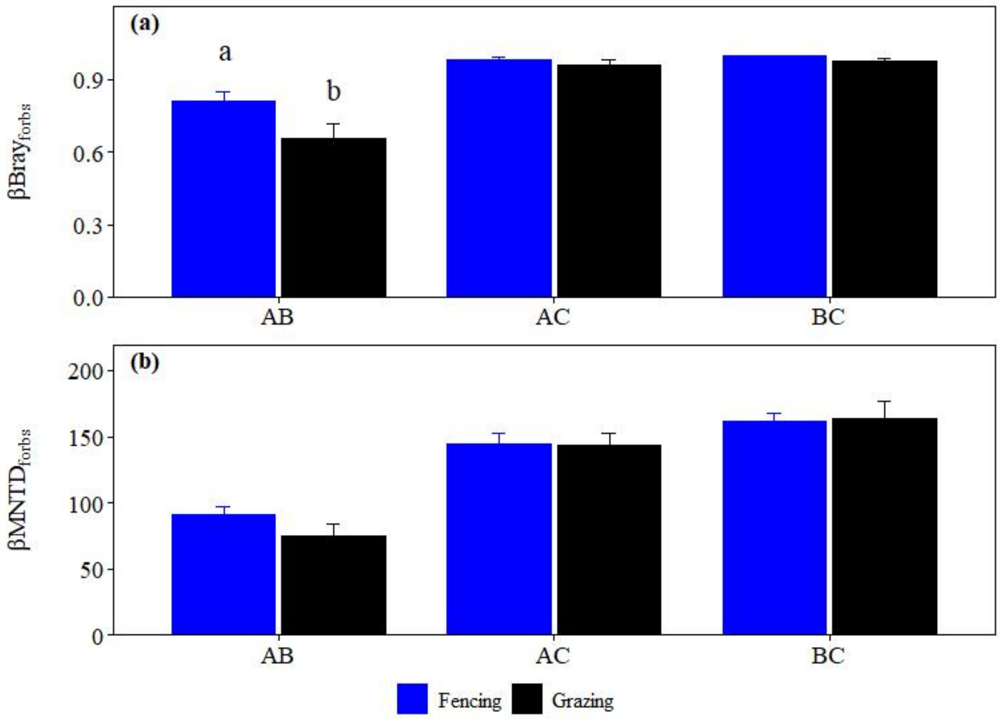

Figure S7 The βBray and βMNTD values (mean ± SE) between any two of the three sites for forbs under fencing and grazing conditions, respectively. AB, AC and BC indicate the βBray and βMNTD between site A and B, between site A and C, and between site B and C, respectively.
